# Predictive simulation of single-leg landing scenarios for ACL injury risk factors evaluation

**DOI:** 10.1371/journal.pone.0282186

**Published:** 2023-03-09

**Authors:** Evgenia Moustridi, Konstantinos Risvas, Konstantinos Moustakas

**Affiliations:** Department of Electrical and Computer Engineering, University of Patras, Patras, Achaia, Greece; Universiti Sains Malaysia, MALAYSIA

## Abstract

The Anterior Cruciate Ligament (ACL) rupture is a very common knee injury during sport activities. Landing after jump is one of the most prominent human body movements that can lead to such an injury. The landing-related ACL injury risk factors have been in the spotlight of research interest. Over the years, researchers and clinicians acquire knowledge about human movement during daily-life activities by organizing complex *in vivo* studies that feature high complexity, costs and technical and most importantly physical challenges. In an attempt to overcome these limitations, this paper introduces a computational modeling and simulation pipeline that aims to predict and identify key parameters of interest that are related to ACL injury during single-leg landings. We examined the following conditions: a) landing height, b) hip internal and external rotation, c) lumbar forward and backward leaning, d) lumbar medial and lateral bending, e) muscle forces permutations and f) effort goal weight. Identified on related research studies, we evaluated the following risk factors: vertical Ground Reaction Force (vGRF), knee joint Anterior force (AF), Medial force (MF), Compressive force (CF), Abduction moment (AbdM), Internal rotation moment (IRM), quadricep and hamstring muscle forces and Quadriceps/Hamstrings force ratio (Q/H force ratio). Our study clearly demonstrated that ACL injury is a rather complicated mechanism with many associated risk factors which are evidently correlated. Nevertheless, the results were mostly in agreement with other research studies regarding the ACL risk factors. The presented pipeline showcased promising potential of predictive simulations to evaluate different aspects of complicated phenomena, such as the ACL injury.

## Introduction

The knee joint is one of the most complex human body anatomical structures. Its stability and functionality during daily life activities are maintained mainly by the articulations, ligaments, menisci and muscles. Among the knee joint ligaments, Anterior Cruciate Ligament (ACL) is of prominent importance, as it acts as a stabilizer by restricting excessive posterior and anterior knee displacement during dynamic movements. This is the reason why ACL rupture is one of the most common knee injuries in competitive sports activities, like football, basketball, or skiing [1]. These sports involve movement patterns of high risk, such as sudden stops, abrupt changes of direction, landing after a jump and deceleration before changing direction [2–4]. These are labeled as non-contact ACL injury conditions.

ACL injury may manifest itself through a complete or partial ligament tear. This condition, as well as the desired post injury activity levels, affect the chosen treatment procedure. Typically, there are two main approaches. The first option involves a basic rehabilitation plan including physiotherapy and bracing support. However, this option requires low levels of activity in the future. On the other hand, if high levels of activity are desired, surgery for ACL restoration is unavoidable. This procedure named as “acl reconstruction” has emerged as the golden standard approach when it comes to ACL injuries [5]. A post-effect of a complete ACL tear is the higher risk of osteoarthritis development on the knee cartilage, especially when the meniscus is also damaged [6, 7]. ACL reconstruction is a complex surgery that depends on a plethora of parameters and requires long periods of rehabilitation, that can span a 6–12 month range [8, 9]. Moreover, graft failures are common and full restoration of knee kinematics is not always achievable. Therefore, complete and accurate knowledge regarding knee joint biomechanics is crucial to develop training strategies that aim to limit ACL injury risk or assist surgery and rehabilitation plans post-injury.

Traditionally, researchers and clinicians acquire knowledge about human movement during daily-life activities by organizing complex *in vivo* studies. In the most common approach, biomechanical studies usually require human motion data that are recorded using dedicated equipment. Examples of such devices are mobile sensors (Inertial Measurement Units or IMUs) and Motion Capture (MoCap) equipment using video technologies and retroreflective markers. The recorded data are further analyzed using dedicated software to estimate joint angles and forces. That way, researchers gain insight into the functionality and response of internal structures such as soft tissues, ligaments and muscles. Estimation of these kinematic and kinetic parameters is also achievable with real-time biofeedback [10]. However, these experiments require dedicated hardware, feature high complexity and costs, and impose physical and psychological challenges to participants. On the other hand, modern biomechanics approaches take advantage of the rapidly increased computational power to develop computer models that mathematically describe all the aspects of the counterpart physical system [11]. The response of these models is numerically evaluated by applying rigid body dynamics, Finite Element Method (FEM) [12, 13] or computational fluid dynamics (CFD), depending on the phenomena under consideration [14]. Combining rigid body dynamics and FEM is also a common route for researchers [15]. Examples of biomechanics application fields using multi-body systems are neuromuscular pathologies [16–18], study of muscle coordination [19–21] and surgery modeling and simulation [22, 23].

### Related work

In this section, we present a review of research studies that follow an experimental and/or simulation approach regarding the identification of ACL injury risk factors during single-leg landings.

One of the most commonly investigated parameter is the height at the start of the drop-landing motion. Several studies have examined how drop height can affect ACL injury risk factors during single-leg and double-leg landings [24, 25]. The importance of hamstring muscles has been noted due to their ability to posteriorly move the tibia bone, and therefore reduce the load on the ACL [26]. Moreover, it has been observed that quadricep and hamstring muscles present greater forces for landings from increased height at time of max vGRF [26]. Additionally, when the Quadriceps/Hamstrings force ratio (Q/H force ratio) is greater than 1, the quadriceps cause the tibia to translate anteriorly which can increase the risk of ACL rupture. On the other hand, when the ratio is less than 1, the increased hamstring muscles contribution cause a posterior tibia movement protecting the excessive and abrupt ACL extension. Furthermore, it has been shown that as the landing height is increased the vertical forces of all ankle, knee and hip joints are also increased [27, 28].

Another risk factor of interest that has been associated with ACL injury is the static lower extremity alignment. This condition refers to the rotational alignment of the knee and hip joints during landings that poses the knee in a valgus posture. More specifically, non-contact ACL rupture includes internal tibia rotation and valgus collapse [29]. It has been stated that dynamic knee valgus is one of the potential biomechanical factors that reduce the capacity to attenuate the impact imposed on the knee joint during landings [30, 31]. Researchers have examined multiple combinations of hip and knee joint kinematics and kinetics during drop-landing. It has been shown that during landings with internally rotated hip, the knee joint experiences greater valgus motion, while the hip rotation moment increases tending to place the knee joint in a valgus configuration [32]. Moreover, stiff landings have been associated with ACL injuries. Lower knee flexion and higher peak vertical Ground Reaction Force (vGRF) during stiff landings were observed, indicating their correlation with high ACL injury risk [33, 34]. Furtherore, a great number of studies examine videos that capture the motion of athletes while ACL injuries occur. A common observation was the hip internal rotation at the injury time instant [35, 36]. Internal hip rotation translates the knee more medial to the Ground Reaction Forces (GRF) vector, thus increasing the moment arm of the force and introducing a greater moment at the knee joint. This further results to an increase of the GRF and higher risk of injury [37]. Additionally, it has been shown that the toe-in landing position is associated with increased hip internal rotation, knee abduction, ankle inversion angles at Initial Ground Contact (IGC) and peak knee IRM, knee AbdM and hip AbdM [38].

Furthermore, several studies investigated the trunk orientation during landings and its association with knee injuries. It has been observed that a trunk in a flexed position leads to knee and hip joint increased flexion, that can limit stiff landings [39–41]. Moreover, the peak of vGRF and the mean quadriceps Electromyography (EMG) amplitude are lower during the flexed trunk landing compared with an extended trunk posture. Regarding right and left lateral trunk bending, it has been found that the knee valgus angle was increased during the left lateral flexion trunk position (when leaning toward the opposite site of the landing side) compared to trunk right lateral flexion position during right-leg landings [42].

Finally, the contribution of the knee surrounding muscles should not be ignored when studying ACL injuries during single-leg landings [25, 43, 44]. Muscle forces contribute to knee joint forces and moments during dynamic movements. A dynamic simulation study with MoCap data revealed the importance of the muscles spanning the knee joint in injuries during the weight acceptance phase of single-leg jump landings [45]. The authors observed greater maximum muscle forces for quadriceps, gastrocnemius and then hamstrings (in decreasing order). They proposed that the elevated quadriceps and gastrocnemius forces can protect from external knee loading, thus, reducing the ACL injury risk.

### Motivation—Contribution

A common aspect of the studies presented in Related work is that they required experimental data, such as MoCap and biosensor measurements (EMG, or sensor implants). Therefore, the demands of “state of the art” technology facilities, equipment and complex experimental setups with multiple trials were unavoidable. In an attempt to overcome these challenges that are inherent in traditional biomechanics approaches, predictive simulations have emerged as a valuable counterpart. The main advantage of these computational approaches is that they allow researchers to predict and study new biological motions, circumventing the inherent barriers of traditional techniques, that were based on experimental data recorded with MoCap equipment. Therefore, multiple simulation studies can be conducted to identify and determine predominant injury factors overcoming the necessity of laborious and demanding experimental setups. Nonetheless, experimental data can still be included in studies to assist or validate the predictive simulation results.

In this work, we propose a predictive simulation approach that allows prediction of a single-leg landing motion without using experimental data. The aim of the presented workflow is to perform multiple case studies of single-leg landings, compare them, and identify factors that could contribute to an ACL injury, thus revealing the potential of modern computational models and simulations for predictive *in silico* trials. In particular, our analysis is divided in three main parts. First, we predict the motion of a single-leg landing using a simplified computational musculoskeletal model. Then, we track the predicted motion using more complex musculoskeletal models. Finally, we predict multiple single-leg landing motions deploying a further complex model and the previously tracked motion as an initial guess. The examined case studies are divided in five main categories. First, we predict landings from various landing heights. Subsequently, we predict single-leg landings with different hip rotation angles. Furthermore, we investigate drop-landings with deviations of the trunk orientation, and in particular the following cases: lumbar flexion and extension, and lumbar medial and lateral bending. Moreover, we predict single-leg landings with different combinations of knee joint agonist and antagonist muscle forces. Finally, we examine the effect of effort on the results of the analysis, since knee stability during single-leg landing can be affected by muscle forces and proprioception.

## Methods

**Fig 1:**
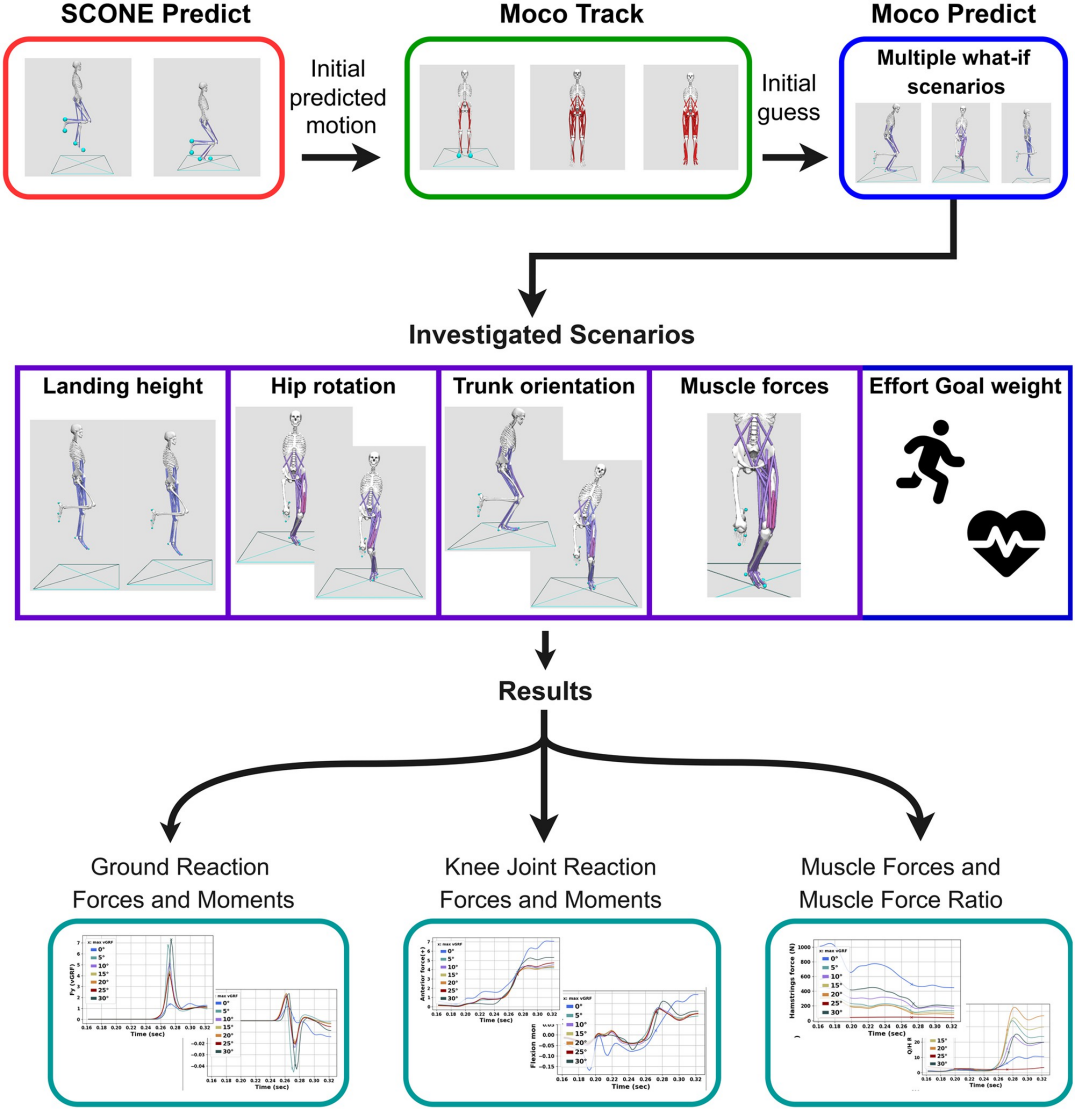
Overview of the proposed simulation pipeline. In this figure, the adopted workflow for this work is presented. In the first layer, the implemented methods and software tools used in this work are presented. The second layer illustrates all the examined scenarios. Finally, in the third layer the measured parameters that are related to ACL injuries during single-leg landings are demonstrated.

In this section, we present the musculoskeletal models that were deployed in the simulations along with the implemented methods of this study. An overview of the proposed workflow is presented in Fig 1. In brief, this pipeline first predicts a single-leg landing motion for a simplified model. Then, it tracks that motion using more complex models. Finally, it predicts multiple what-if scenarios of landing motions, where the previously tracked motion is used as an initial guess for the optimization algorithm. These steps are demonstrated in the first layer of Fig 1. In the following two layers of Fig 1 we illustrate all the examined scenarios and the measured parameters associated with ACL injuries based on literature review, respectively.

To eliminate ambiguity, a description of the terms *Track* and *Predict* is crucial before proceeding with the detailed description of the adopted methodology. These terms refer to the type of optimization problem we solve. *Track* is a short term for motion tracking. In tracking simulations, the errors between model kinematics (motion and muscle or other actuator controls) and reference data from an observed motion are minimized. Tracking simulations provide a new motion allowing deviations from the experimental motion data. A common application of tracking studies is the creation of initial guesses for predictive optimizations. On the other hand, *Predict* is a short term for predictive simulations that are conducted to predict new motions based on defined goals and constraints.

### Musculoskeletal modeling

**Fig 2:**
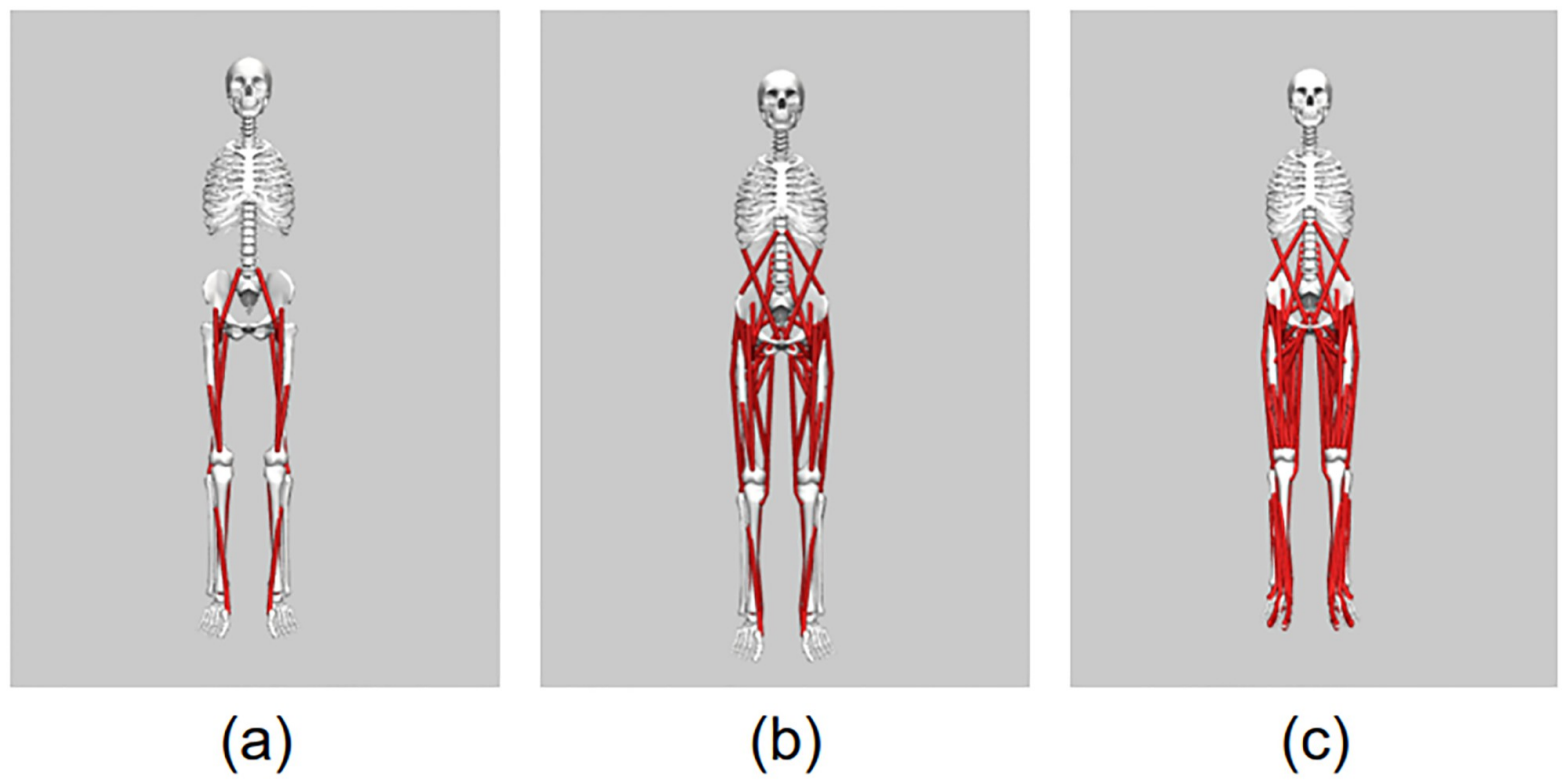
The three musculoskeletal models that are used throughout the simulations of this work. They appear in order of increasing complexity from left to right. (a) The “Human0916”, (b) “Gait2354”, and (c) “Gait2392” model.

The backbone of the present work is comprised of the musculoskeletal models developed and analyzed through the OpenSim software [46]. For the purposes of this research study we utilized three models with augmented complexity in terms of Degrees of Freedom (DoFs) and muscle-tendon actuators. All models correspond to a subject of 1.8 m height and 75.16 kg weight. The less complex model is named “Human0916” and is a 2D planar reduced gait model with 9 Degrees of Freedom (DoFs) and 16 muscle-tendon actuators. Also, it includes a representation of the foot-ground contact mechanism. The other two models are named “Gait2354” and “Gait2392” and they feature 23 DoFs enabling 3D kinematics. The former consists of 54 musculotendon units while the latter has 92 musculotendon actuators for modeling 76 muscles that span the joints of the lower limbs and pelvis. In both models, the knee structure is represented by a hinge joint. Therefore, the only unlocked DoF is knee flexion while the remaining DoFs are prescribed based on experimental data [47]. The three musculoskeletal models are presented in order of increasing complexity from left to right in Fig 2.

### Predict single-leg landing motion with SCONE

The aim of the first predictive simulation was to obtain an initial single-leg landing motion using a simplified model. This motion will serve as an initial set of state variables for the upcoming studies. For this purpose, we deployed the Scone software, an optimization and control computational framework for predictive simulations dedicated to study biological motion [48]. SCONE builds on OpenSim and is based on shooting methods for transcribing an optimal control problem. The control problem is described through a defined scenario that includes several components, such as the musculoskeletal model, a controller for determining excitation, and several measures that serve as constraints.

**Fig 3:**
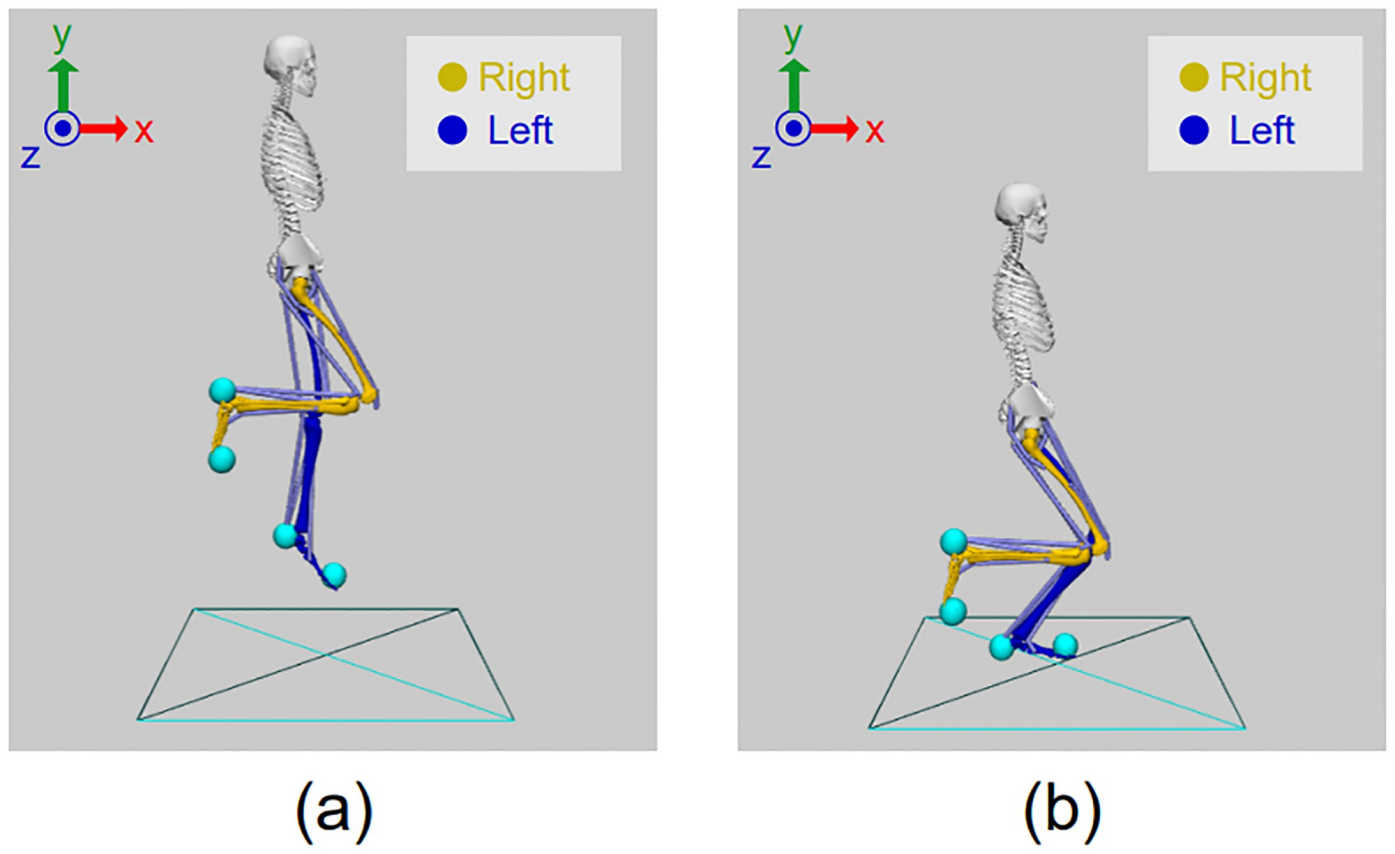
Initial and final states of the singles-leg landing motion. (a) The initial and (b) the final states of the “Human0916” musculoskeletal model for prediction of single-leg landing motion in SCONE. The reference axis of the defined initial joint angles are about the z-axis of each parent frame that coincides with the global z-axis, since the adopted model is planar.

In the scope of this work, we utilized the “Human0916” musculoskeletal model, described in Musculoskeletal Modeling. Initially, the model was imported to OpenSim to apply an initial configuration and define the desired initial and final states of the single-leg landing motion, as demonstrated in Fig 3.

The desired landing height was set to 30 cm. This value is similar to other studies evaluating single-leg landings [24, 26, 27]. To achieve this, the DoF corresponding to the pelvis vertical translation was set to 1.25 m. Subsequently, we modified the DoFs of both lower-limbs to achieve a single-leg landing with the left foot. For the initial state of that motion, the left hip flexion angle was set to 5°, the left knee flexion angle was set to 12 and the left ankle angle was set to 34. Furthermore, the DoFs of the right-limb were locked to specific values to prevent it from touching the ground at contact phase. The right hip flexion was locked at 25, and the right knee and hip angles were locked at 120. Moreover, all DoFs of the upper body were set at their default values apart from the pelvis vertical translation as previously mentioned.

Moreover, we included an already implemented reflex controller (with a fixed step size equal to 0.005) which contained entries that simulated proprioceptic and vestibular reflexes [49]. Finally, we added a composite measure that described the desired task. This set of distinct measures serves as the objective function and enforces the following penalties:

- **A parameter that checks if the model falls below a specified value:** This measure evaluates the ratio between the Center of Mass (COM) height (*com*_*h*_) to the initial state ($com_{h_{in}}$), and checks if it lower than the expected threshold:
  $$\begin{array}{c} \frac{com_{h}}{{com}_{h_{in}}}<th \end{array}$$ (1)
  The penalty value for the stability is given by the next formula:
  $$\begin{array}{c} p_{s}=w_{s}\cdot \left(\frac{t_{max}-t_{sim}}{t_{max}}\right) \end{array}$$ (2)
  where *th* is the threshold for COM height, *t*_*max*_ is the maximum duration of the simulation, *t*_*sim*_ is the time after which the simulation is terminated, and *w*_*s*_ the weight of the stability penalty.
- **Penalties for exceeding desired joint ranges**. This penalty evaluates the differences between the desired and actual joint angles and is formulated as follows:
  $$\begin{array}{c} p_{j}=w_{j}\cdot \left|jR_{m}-jR_{t}\right| \end{array}$$ (3)
  where *w*_*j*_ is the weight of the penalty, *jR* is the joint range, “m” refers to the actual values recorded in simulation, and “t” to the desired angles.
- **A penalty minimizing the overcoming of defined GRF ranges:** Calculate GRF and adds a penalty (*p*_*GRF*_) when these are below or above certain thresholds.

Thus, the objective function (or cost function) is given by the summation of all components of the measures set:

$$\begin{array}{c} cost=p_{s}+p_{j}+p_{GRF} \end{array}$$ (4)

The result of this simulation was a single-leg landing motion that was used for the upcoming simulations with the more complex musculoskeletal models, as it will be presented in the upcoming subsection. To aid reproducibility, simulation settings are presented in more detail in S1 File.

### Track single-leg landing motion with Moco

In this section, we describe the adopted approach for predicting single-leg landing using the previously predicted motion (Predict single-leg landing motion with SCONE) and the 3D OpenSim models of higher complexity (Musculoskeletal Modeling). The objective was to create an initial guess of a single-leg landing motion that would serve as a baseline for all upcoming case studies. Towards this direction, we utilized the OpenSim Moco software tool. Moco is a software toolkit for optimizing the motion and control of musculoskeletal systems [50]. It solves problems of trajectory optimization based on the robust direct collocation method that exhibits lower computational cost with robust accuracy compared to the traditional shooting methods [51].

Among other, Moco offers the ability to track a recorded or predicted motion and adjust it based on user-defined constraints. Taking advantage of this, we created two Moco tracking studies, with a twofold purpose. First, we created an initial guess for the upcoming optimization studies. Secondly, we chained three distinct simulations with each one capitalizing on the optimal solution of the previous one and featuring a musculoskeletal model of higher complexity.

**Fig 4:**
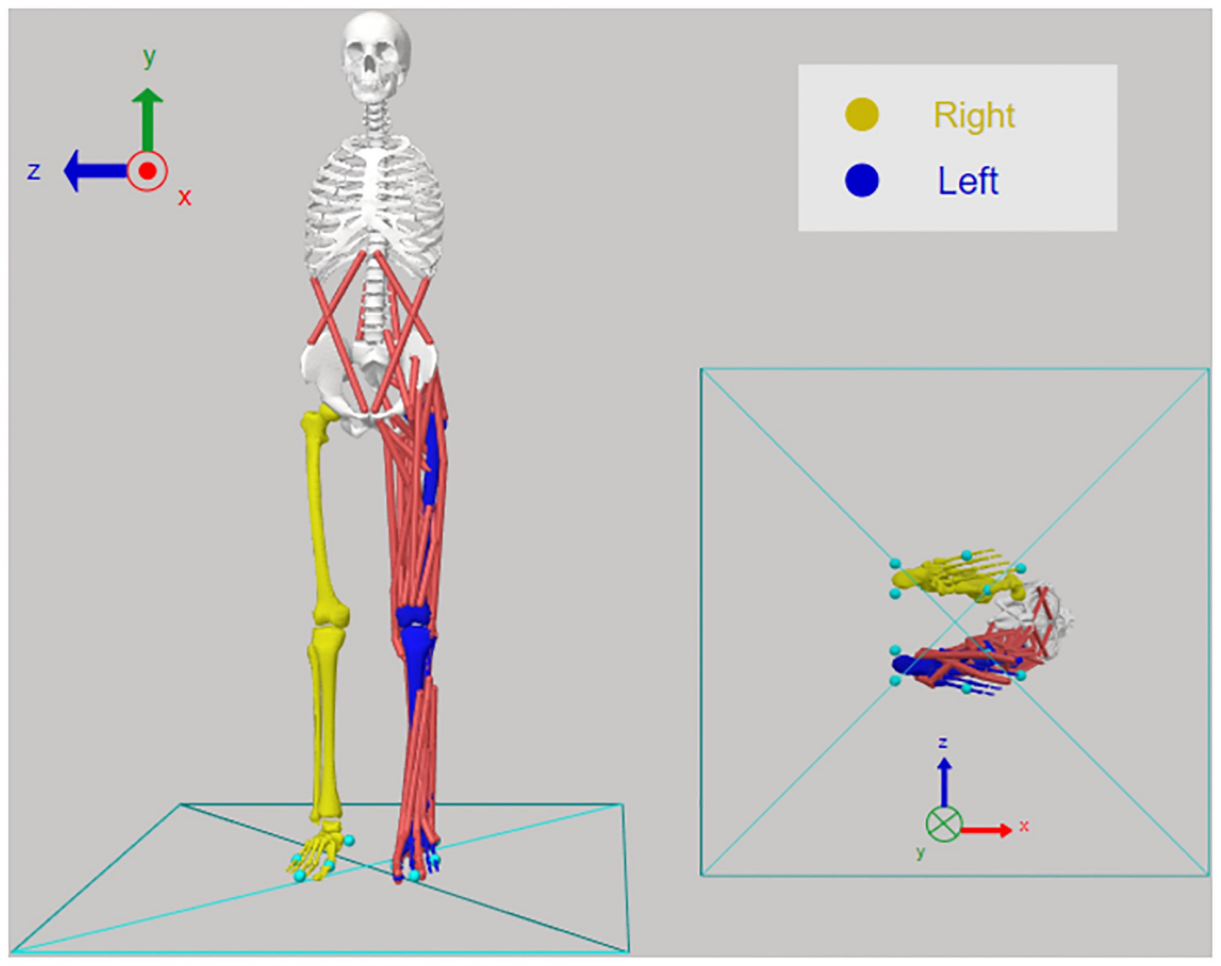
Schematic diagram of the “Gait2392” musculoskeletal model. Schematic diagram of the “Gait2392” model with only the left side muscles. Foot-ground contact was modeled with five contact spheres per foot.

Initially, we extended and optimized both “Gait2354” and “Gait2392” models. Specifically, foot-ground interaction was modeled using an OpenSim compliant contact force model [52]. Five spheres were placed under each foot of the model to simulate the interaction of the foot and the ground. One sphere was placed under the toes and four under the hind-foot, as demonstrated in Fig 4. The values for parameters like stiffness, dissipation and friction were assigned based on other research studies [52] and are presented in the S1 File. Moreover, the position and total number of the contact spheres were adopted by a study for simulating a standing vertical jump [53].

Also, it should be mentioned that in order to speed up and simplify the simulations, all muscles of the right lower limb were omitted, since we were interested only in studying landing motion with the left lower-limb (Fig 4). The DoFs of the right part were set at the same values of the initial state of SCONE pipeline and kept locked throughout all simulations. Finally, ideal actuators with low optimal forces were appended to all DoFs to assist muscles and simulation convergence.

Concerning the three chained simulations, these are the previously described SCONE simulation (Predict single-leg landing motion with SCONE), and two tracking studies using the Moco tool. In both of these Moco simulations, we created a “Moco Track” tool instance and a solver that described the problem and the desired solver accordingly. The “Moco Track” tool defines an objective function for tracking joint angles, actuator activation and other parameters of interest. It is formulated as the squared difference between a reference state variable value and a state variable value, summed over the state variables for which a reference is provided. All the additional simulation settings are described in detail in S1 File. The main differences between the two simulations were the musculoskeletal model and the motion we aimed to track. For the first simulation we used the “Gait2354” model and the predicted motion from SCONE. At the end of the simulation we acquired a prediction for the state of additional DoFs that were not available in “Human2016”. The output of this simulation was fed to the second simulation where the most complex model “Gait2392” was used. The intermediate simulation was used to speed up simulation time and assist in convergence, since now only the additional muscle states of the third model had to be predicted without an initial guess for their values.

The output of the second tracking study was again a single-leg landing motion, but this time it included states and forces for a greater number of DoFs, as we used a more complex musculoskeletal model. The resulting motion was used as an initial guess for the upcoming simulations.

### Predict single-leg landing motion with Moco

In this section, we describe the adopted methodology for conducting multiple simulations that predict what-if scenarios based on the acquired initial guess. The initial guess is the single-leg landing motion acquired from the final step of Track single-leg landing motion with Moco. This initial guess describes the evolution of each model state throughout the predicted single-leg landing. These state variable trajectories were used again in Moco to conduct a compendium of studies where deviations on body posture and muscle forces were applied and a new single-leg landing motion was predicted. Throughout all studies we used the most complex model, “Gait2392”, and a Moco effort goal as the objective function. This goal is used for minimizing the sum of the absolute value of the controls raised to an exponent. In our case, the exponent was set to 2, to achieve minimization of squared muscle activation. The goal is computed by the following formula:

$$\begin{array}{c} \frac{1}{d}\int_{t_{i}}^{t_{f}}\sum_{c\in C}^{}w_{c}{\left|x_{c}\left(t\right)\right|}^{p}dt \end{array}$$ (5)

where:

*d*: the displacement of the system,

*C*: the set of the control signals,

*w*_*c*_: the weight for the control C,

*x*_*c*_: the control signal c, and

*p*: the exponent.

Moreover, to execute all the what-if scenarios we assigned suitable bounds for specific DoFs for the initial and final state of the motion for each scenario. Based on modifications on specific DoFs bounds and on simulation properties we created multiple scenarios which are described in detail in Case studies. Details about the simulation setup and the ranges of each state parameter are included in S1 File.

### Case studies

In this section, we present all the scenarios that were examined using the proposed framework. Initially, we created studies for predicting single-leg landing motions from a landing height of 30, 35, 40, 45, 50 and 55’cm. The objective was to identify the effect of landing height on the prominent ACL injury risk factors. This was achieved by adjusting the pelvis joint vertical position of the “Gait2392” for the initial state of each scenario. The ranges for all other DoFs remain identical for all scenarios.

Additionally, we examined landings with different values of hip rotation for the landing lower limb. This parameter is related to knee valgus position, which appears as a potential ACL injury risk factor. We examined cases where the hip was kept internally or externally rotated for the entire simulation. In total, we created 13 studies, 6 for hip internal rotation, 6 for hip external rotation and 1 for no hip rotation. The examined angles for hip external and internal rotation were 5°, 10°, 15°, 20°, 25° and 30°.

Subsequently, we predicted single-leg landings that featured different values of the trunk orientation. This was achieved through modifying the two lumbar joint DoFs, namely flexion and extension and right and left bending. The assessed flexion angles were: 5°, 10°, 15°, 20°, 25° and 30°, while for extension were: 5°, 10°, 15° and 20°. Moreover, the angles studied for right and left bending were: 5°, 10°, 15°, 20°, 25° and 30°. The upright position with no flexion, bending or rotation was also considered in our study.

Furthermore, we examined permutations of maximum isometric force for the knee joint agonists and antagonists muscle groups that affect the ACL injury indicators. Muscle fatigue is defined as the decrease in maximum isometric force [54]. Towards this objective, we edited the values of max isometric forces to simulate weakness and strength of specific muscle groups, namely the quadriceps and the hamstrings. To simulate strong muscles we increased maximum isometric force by 35% and to simulate weak muscles we reduced it by 35%. These percentage values are inside the limits of published musculoskeletal studies that investigate the sensitivity of muscle parameters, such as the maximum isometric force [55–57].

During the last case scenario, we examined how the Moco effort goal (minimization of squared muscle activations, 5) affects the outcome of the study. More specifically, the purpose of this case study was to better understand how activation of muscle forces may affect GRF and which weight factor would lead to estimated GRF values close to those reported in literature. The following goal weight values were investigated: 0, 0.1, 0.2, 0.5, 1, 2, 5 and 10.

An overview of the investigated scenarios is presented in Table 1. Additional information about all scenarios and the assigned bounds on the DoFs can be found on S1 File. Finally, for all the previously presented case studies we extracted specific parameters of interest. These were the muscle forces, GRF, Knee Joint Reaction Forces (kJRF), Knee Joint Reaction Moments (kJRM) and the Q/H force ratio.

**Table 1:** Overview of the investigated scenarios to identify ACL injury risk factors during single-leg landings.

Case studies		
Case	Variable	Values
Landing Height	Pelvis vertical height	30, 35, 40, 45, 50, 55
Hip	Internal Rotation	5, 10°, 15°, 20°, 25°, 30°
	External Rotation	5, 10°, 15°, 20°, 25°, 30°
Trunk	Flexion	5, 10°, 15°, 20°, 25°, 30°
	Extension	5, 10°, 15°, 20°
	Right Bending	5, 10°, 15°, 20°, 25°, 30°
	Left Bending	5, 10°, 15°, 20°, 25°, 30°
Muscle forces	Quadriceps *F*_0_	1.35%*F*_0_, *F*_0_, 0.65%*F*_0_*
	Hamstrings *F*_0_	1.35%*F*_0_, *F*_0_, 0.65%*F*_0_
Effort Goal	Weight	0, 0.1, 0.2, 0.5, 1, 2, 5, 10

^†^ *F*_0_ denotes the maximum isometric force of each muscle.

## Results

In this section, we present the results regarding the risk factors associated with ACL injuries for each case study. These factors are the hip kinematics, vGRF, quadricep and hamstring muscle forces and Q/H force ratio for the landing lower limb. Also, we include kJRF and kJRM for the knee joint of the landing leg. The kinematics are presented in degrees, the GRF, Joint Reaction Forces (JRF) and Joint Reaction Moments (JRM) are presented with normalized values to Body Weight (BW) of the musculoskeletal model. The muscle forces are presented in Newtons.

### Landing height case

In this subsection, we present results concerning the predictive simulations of landing motions from multiple landing heights (30, 35, 40, 45, 50 and 55 cm). The results clearly indicate the association of the landing height with the assessed ACL injury risk factors.

**Fig 5:**
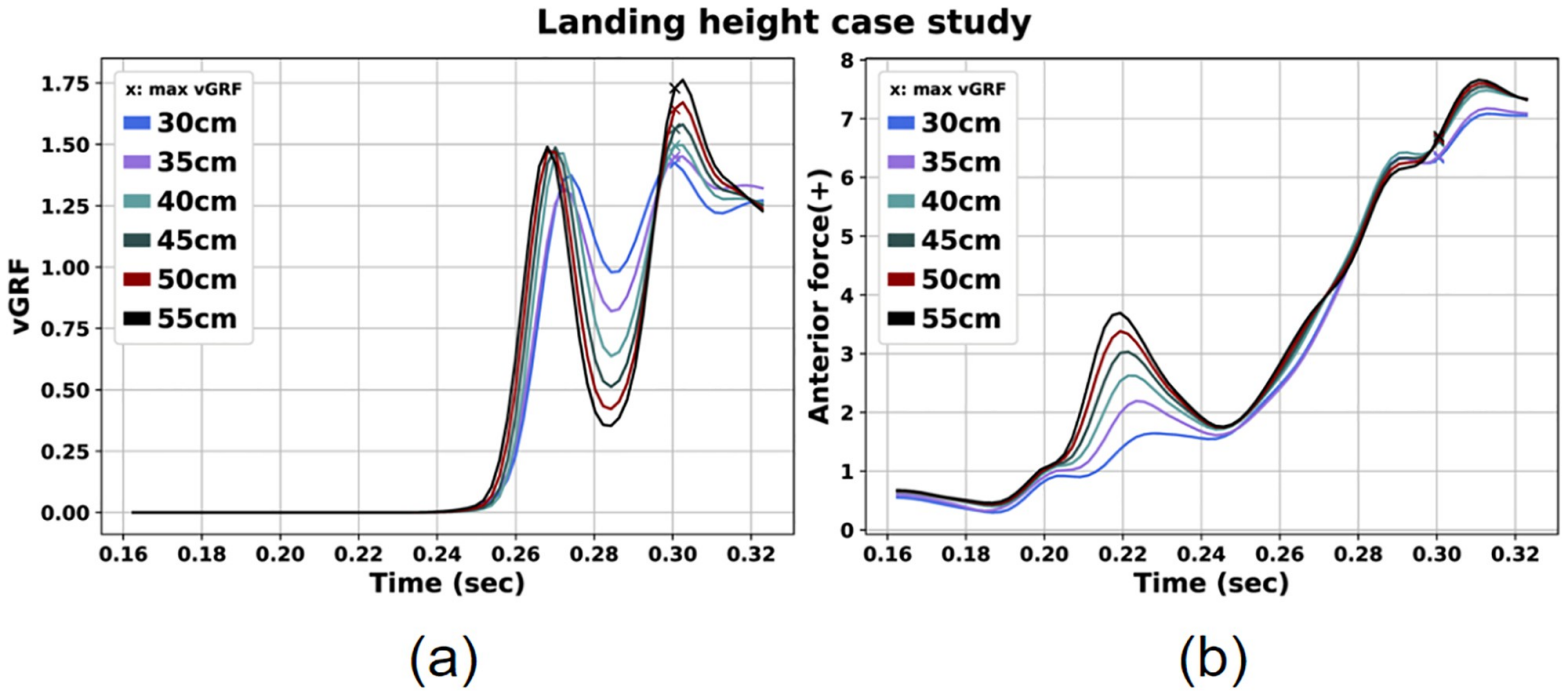
Vertical GRF (a) and, knee joint AF (b) for the landing height case study.

Initially, we observe that as the landing height was increased the peak landing forces (GRF) were also increased, as illustrated in Fig 5. Also, the AF reaches a first peak after IGC and then a greater one after the max vGRF time instance for all cases (Fig 5). Both peaks are greater as the landing height is increased. Nevertheless, at max vGRF no large differences can be observed for the AF among the scenarios. Still, if we take a closer look at the peak vGRF time instance (Table 2), we can observe greater AF values for the landings from greater heights. In Table 2, we present the vGRF, AF, AbdM and Q/H force ratio at the peak vGRF moment for all scenarios. Regarding AbdM, we note larger values for landings from increased landing heights. On the contrary, Q/H force ratio obtains lower values for the landings from 55 and 50 cm.

**Table 2:** Vertical GRF, AF, AbdM and Q/H force ratio at peak vGRF time instance for multiple drop-landing heights.

Height(cm)	vGRF	AF(+)	AbdM(+)	Q/H_ratio
30	1.436	6.300	0.021	9.243
35	1.467	6.334	0.014	9.167
40	1.523	6.577	0.023	9.250
45	1.604	6.612	0.025	9.083
50	1.698	6.652	0.029	8.951
55	1.797	6.699	0.033	8.850

### Hip rotation case

Next, we present the results concerning single-leg landings for different values of internal and external hip rotation applied to the landing lower limb. These angles were set at 0°, 5°, 10°, 15°, 20°, 25° and 30° for both cases.

**Fig 6:**
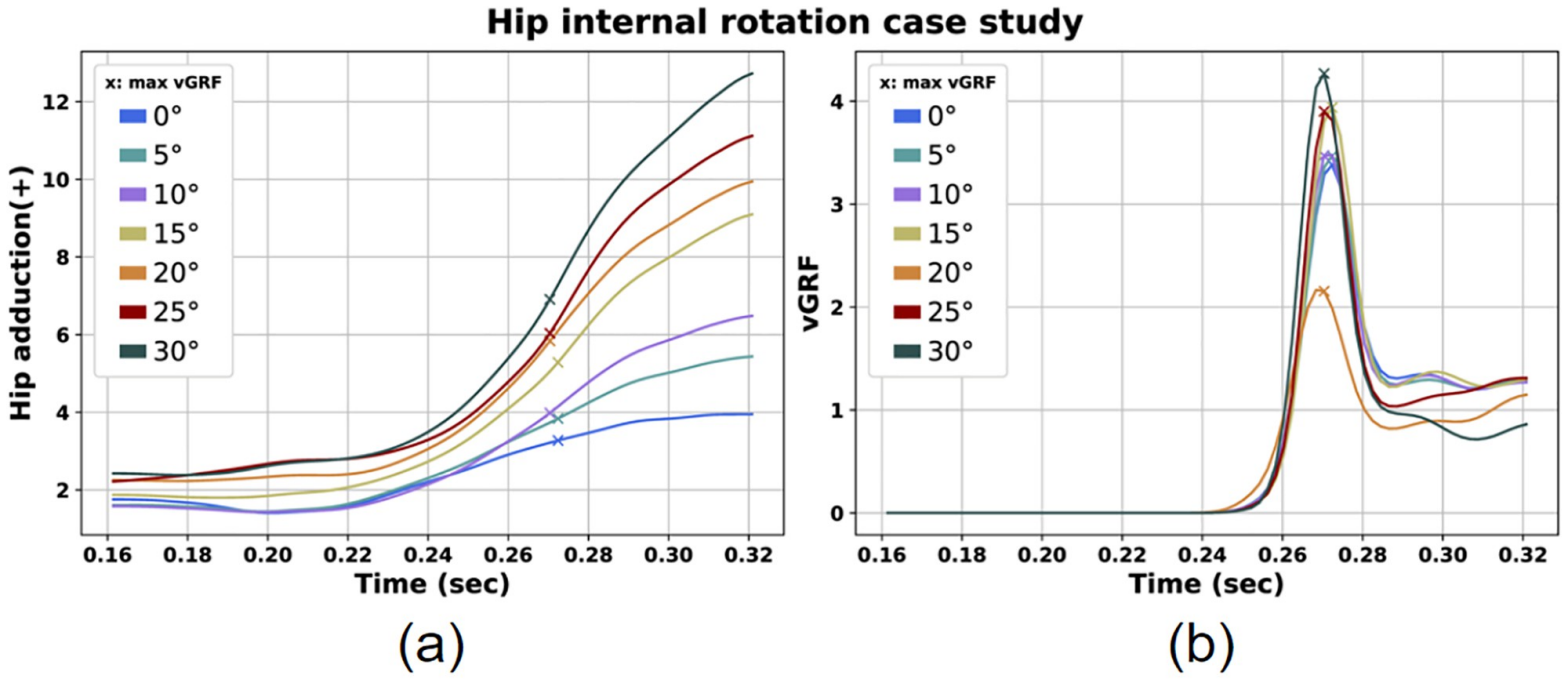
Hip adduction (a) and vGRF (b) for the hip internal rotation case study.

First, we observed that as we imposed greater hip internal rotation angles, the hip adduction was also increased (Fig 6 (a)). Moreover, it is noticeable from Fig 6 (b) that the minimum vGRF peak occurs for the 20° case (2.234 times the BW). Although, for that scenario we detected greater AbdM at peak vGRF (0.044 times BW). Nevertheless, for the 0° hip internal scenario the peak vGRF was 3.602 times BW while AbdM was 0.020. As the hip internal rotation was increased further, peak vGRF was also increased (reaching the value of 4.658 times BW for the 30° scenario). These values are presented in Table 3.

**Table 3:** Vertical GRF, knee JRF and JRM and Q/H force ratio at peak vGRF time instance for various hip internal rotation angles.

Angle(°)	vGRF	AF(+)	MF(+)	AbdM(+)	IRM(-)	Q/H_ratio
0	3.602	3.927	0.231	0.020	-0.034	12.264
5	3.722	3.982	0.207	0.031	-0.022	9.911
10	3.795	3.578	0.208	0.021	-0.030	11.007
15	4.310	3.787	0.231	0.028	-0.027	12.775
20	2.234	3.605	0.201	0.044	-0.043	7.016
25	4.417	3.809	0.213	0.027	-0.019	9.860
30	4.658	4.068	0.310	0.052	-0.028	13.880

**Fig 7:**
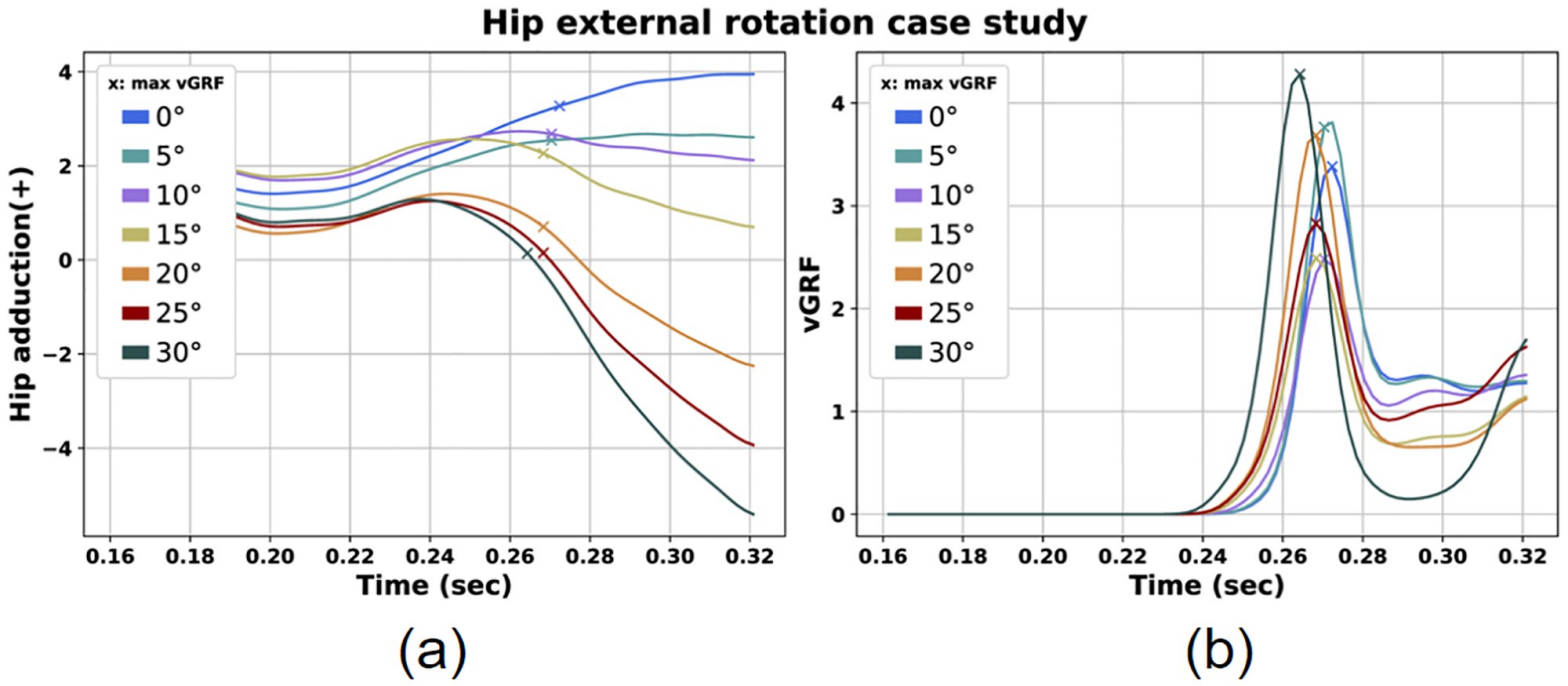
Hip adduction (a) and vGRF (b) for the hip external rotation case study.

Regarding the predicted landing motions with externally rotated hip we can spot the lowest vGRF peaks for the 10° and 15° hip external rotation scenarios (Fig 7 (b)). Also, at peak vGRF time instance the 0° hip rotation scenario presents greater AF, MF, AbdM and IRM compared with the scenarios featuring externally rotated hip (Table 4). Moreover, we can see that for increased, but not excessive external hip rotation angles, the AF is noticeably smaller converging to the same conclusion. Last but not least, we can notice that as the hip external rotation angle is increased, the Q/H force ratio decreases.

**Table 4:** Vertical GRF, knee JRF and JRM and Q/H force ratio at peak vGRF time instance for various hip external rotation angles.

Angle(°)	vGRF	AF(+)	MF(+)	AbdM(+)	IRM(-)	Q/H_ratio
0	3.602	3.927	0.231	0.020	-0.034	12.264
5	4.108	3.554	0.129	0.005	-0.024	10.065
10	2.593	3.486	0.158	0.002	-0.027	7.399
15	2.584	3.167	0.088	-0.003	-0.012	6.141
20	3.888	3.069	-0.005	-0.002	-0.010	6.307
25	2.924	3.203	0.119	0.010	-0.033	7.250
30	4.501	3.298	-0.046	-0.008	-0.009	4.382

### Trunk orientation case

In this section, we present the results regarding single-leg landings corresponding to different trunk orientations.

Initially, we examined landings with the upright position and landings with lumbar flexion and extension. The estimated risk factors are presented in Tables 5 and 6 for lumbar flexion and extension, respectively. The rows containing the 0° angle correspond to the upright posture. We can observe that when the trunk is leaning forward at 25° the peak vGRF is lower, following by the 30°, and 10° inclinations. However, the lowest vGRF value is observed for the case of the upright posture landing. Also, for the case of 25° trunk flexion, AbdM and IRM are lower compared with the other trunk flexion angles.

**Table 5:** Vertical GRF, knee JRF and JRM and Q/H force ratio at peak vGRF time instance for multiple lumbar flexion angles.

Angle(°)	vGRF	AF(+)	CF(-)	AbdM(+)	IRM(-)	Q/H_ratio
0	1.383	3.756	-7.761	0.006	0.000	6.275
5	6.571	3.862	-12.160	0.094	-0.021	12.713
10	4.485	3.731	-10.090	0.077	-0.014	19.775
15	7.647	4.245	-13.329	0.105	-0.032	59.534
20	7.892	3.829	-13.372	0.132	-0.023	13.545
25	3.674	3.899	-10.024	0.072	-0.013	10.211
30	4.139	3.241	-9.421	0.093	-0.008	13.440

**Table 6:** Vertical GRF, knee JRF and JRM and Q/H force ratio at peak vGRF time instance for multiple lumbar extension angles.

Angle(°)	vGRF	AF(+)	CF(-)	AbdM(+)	IRM(-)	Q/H_ratio
0	1.383	3.756	-7.761	0.006	0.000	6.275
5	1.401	3.793	-7.835	0.007	-0.001	6.391
10	6.403	4.988	-13.681	0.118	-0.036	32.214
15	8.198	3.507	-12.850	0.082	-0.029	30.940
20	4.593	3.800	-9.908	0.074	-0.028	41.636

Regarding landings with the trunk leaning backwards, as a general remark, we observe greater vGRF compared to the upright position as the lumbar extension angle is increased. Additionally, Q/H force ratio, CF, AbdM and IRM are greater at time of maximum vGRF for the cases of landings with the trunk in a backwards leaning posture.

Subsequently, we investigated the effect of trunk bending. We observe that greater AbdM values occur as the right or left bending angle is increased compared with the neutral position, that would introduce an increased knee valgus angle (Tables 7 and 8)). The largest peak vGRF occur when bending towards the opposite to the landing leg direction with a value of 8.632 times the BW.

**Table 7:** Vertical GRF, knee JRF and JRM and Q/H force ratio at peak vGRF time instance for multiple trunk right bending angles.

Angle(°)	vGRF	AF(+)	AbdM(+)	IRM(-)	Q/H_ratio
0	1.383	3.756	0.006	0.000	6.275
5	1.387	3.729	0.012	0.002	6.232
10	1.413	3.754	0.018	0.003	6.322
15	8.632	3.397	0.101	-0.026	37.554
20	7.432	4.794	0.153	-0.021	17.024
25	6.131	4.176	0.131	-0.032	23.210
30	4.515	3.396	0.127	-0.004	14.284

**Table 8:** Vertical GRF, knee JRF and JRM and Q/H force ratio at peak vGRF time instance for for multiple trunk left bending angles.

Angle(°)	vGRF	AF(+)	AbdM(+)	IRM(-)	Q/H_ratio
0	1.383	3.756	0.006	0.000	6.275
5	6.787	3.645	0.078	-0.028	18.631
10	5.091	3.810	0.067	-0.017	15.568
15	4.378	3.594	0.058	-0.022	24.773
20	4.224	3.512	0.055	-0.020	27.775
25	4.088	3.552	0.056	-0.021	2.095
30	7.309	4.135	0.085	-0.008	12.901

### Permutations of knee joint agonists and antagonists muscles case

**Fig 8:**
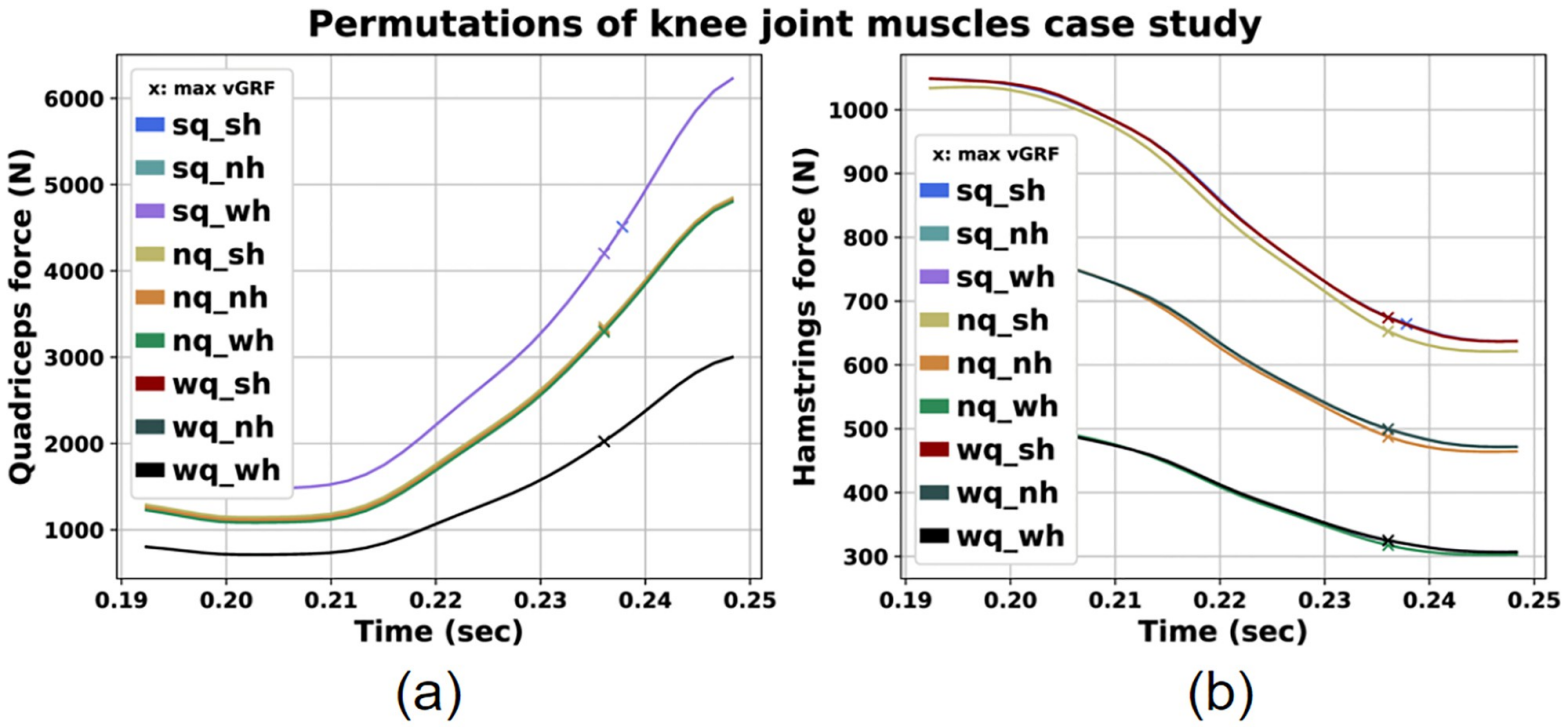
Quadriceps (a) and Hamstrings (b) force for the permutations of the knee joint agonists and antagonists muscles case study.

**Fig 9:**
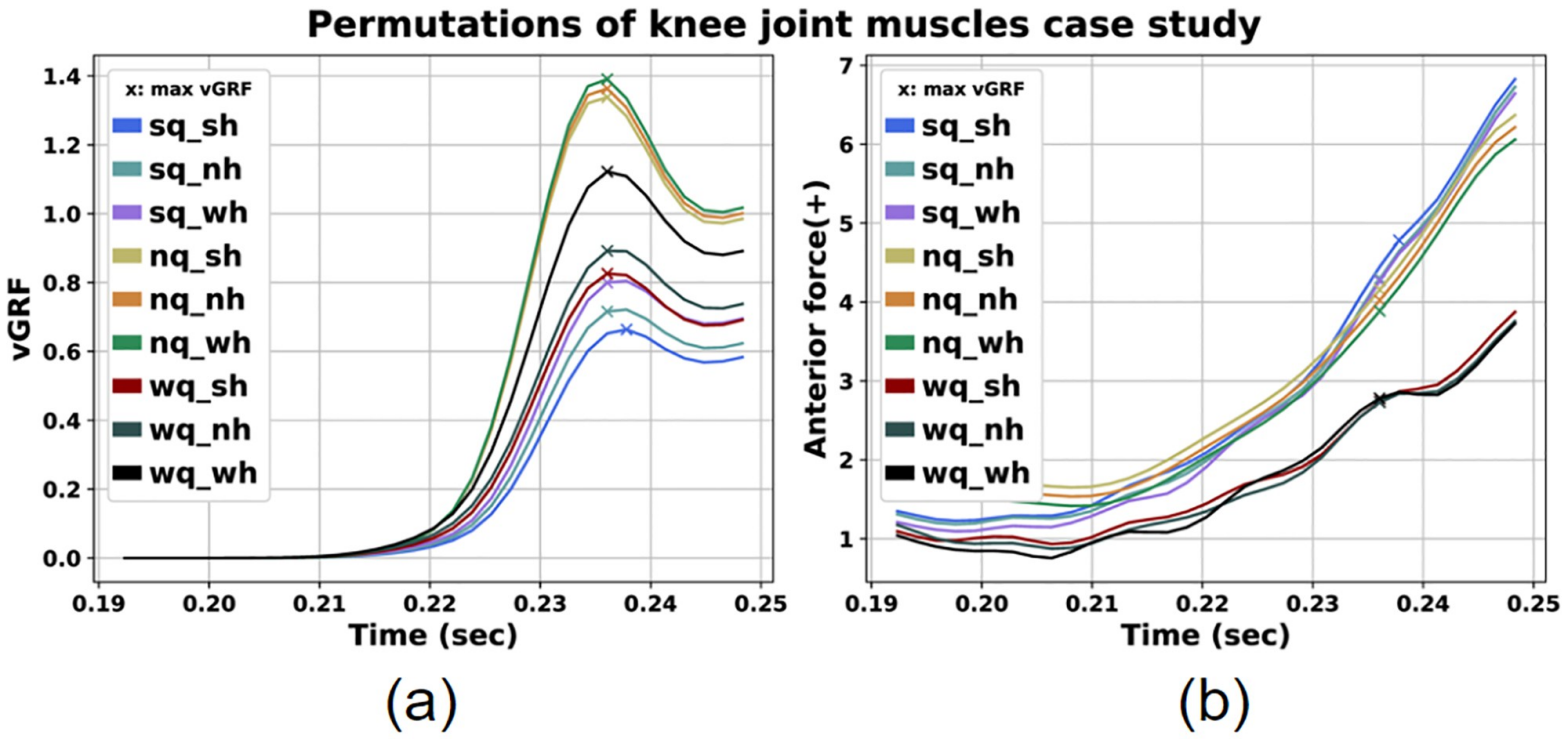
Vertical GRF (a) and AF (b) for the permutations of the knee joint agonists and antagonists muscles case study.

In this subsection, we investigate how permutations of max isometric force for the knee joint agonist and antagonist muscles affect the investigated ACL injury risk factors. The different combinations of normal, weak and strong quadricep and hamstring muscles are presented in Table 9. The results for the muscle forces, vGRF and AF are presented in Figs 8 and 9, respectively. The values of these parameters at peak vGRF time instance are presented in Table 10.

**Table 9:** Demonstration of the nine cases with different combinations of normal, weak and strong quadricep and hamstring muscles.

Case	Case explained	Quadriceps	Hamstrings
sq_sh	strong quadriceps, strong hamstrings	1.35%*F*_0_ ^†^	1.35%*F*_0_
sq_nh	strong quadriceps, normal hamstrings	1.35%*F*_0_	*F*_0_
sq_wh	strong quadriceps, weak hamstrings	1.35%*F*_0_	0.65%*F*_0_
nq_sh	normal quadriceps, strong hamstrings	*F*_0_	1.35%*F*_0_
nq_nh	normal quadriceps, normal hamstrings	*F*_0_	*F*_0_
nq_wh	normal quadriceps, weak hamstrings	*F*_0_	0.65%*F*_0_
wq_sh	weak quadriceps, strong hamstrings	0.65%*F*_0_	1.35%*F*_0_
wq_nh	weak quadriceps, normal hamstrings	0.65%*F*_0_	*F*_0_
wq_wh	weak quadriceps, weak hamstrings	0.65%*F*_0_	0.65%*F*_0_

^†^ *F*_0_ denotes the maximum isometric force of each muscle.

**Table 10:** Knee vGRF, AF, Quadricpes force, Hamstrings force and Q/H ratio at peak vGRF time instance for scenarios of normal, weak and strong knee joint agonist antagonist muscles.

Case	vGRF	AF(+)	Quad Force	Ham Force	Q/H_ratio
sq_sh	0.673	4.835	4508.183	663.738	6.792
sq_nh	0.731	4.333	4210.783	499.288	8.434
sq_wh	0.815	4.316	4210.184	324.788	12.963
nq_sh	1.362	4.168	3360.484	652.619	5.149
nq_nh	1.388	4.035	3332.694	487.502	6.836
nq_wh	1.417	3.902	3305.902	317.691	10.406
wq_sh	0.841	2.781	2027.969	674.048	3.009
wq_nh	0.909	2.774	2028.036	499.098	4.063
wq_wh	1.141	2.829	2028.897	325.101	6.241

We can observe that variation of the maximum isometric force for these two muscle groups affects all simulation results. As illustrated in Fig 8, the scenarios with strong hamstrings resulted in greater hamstrings force. The same holds true for the cases with strong quadriceps.

Moreover concernig GRF, we detect lower vGRF peaks for the scenarios with strong quadricpes (*sq*_*sh*, *sq*_*nh*, *sq*_*wh*), where the lowest peak corresponds to the scenario with both strong hamstrings and quadriceps (Fig 9). Regarding AF, we observe lower values at peak vGRF time instance for the scenarios with weak quadriceps regardless of hamstrings force variation.

A notable observation is that the scenarios with strong quadriceps (*sq*_*sh*, *sq*_*nh*, *sq*_*wh*) exhibit the lower vGRF, but also the greatest AF at peak vGRF time instance compared to other cases. On the contrary, for the cases with weak quadriceps (*wq*_*sh*, *wq*_*nh*, *wq*_*wh*) we can observe greater vGRF peaks and lower AF values at peak vGRF moment.

### Moco control goal weight case

Finally, we investigated how the Moco effort goal that corresponds to minimization of the squared muscle activations as mentioned in Predict single-leg landing motion with Moco, affects the simulation results. The results are presented in Table 11.

**Table 11:** Vertical GRF, AF, Q/H force ratio at peak vGRF time instance for the Moco Control goal weight case.

Case	vGRF	AF(+)	Q/H_ratio
0	1.967	4.956	3.724
0.1	4.499	4.002	11.544
0.2	7.779	3.604	74.678
0.5	11.116	3.225	78.301
1	11.479	3.235	42.970
2	11.113	3.143	77.616
5	11.043	3.141	69.774
10	10.964	3.122	72.103

**Fig 10:**
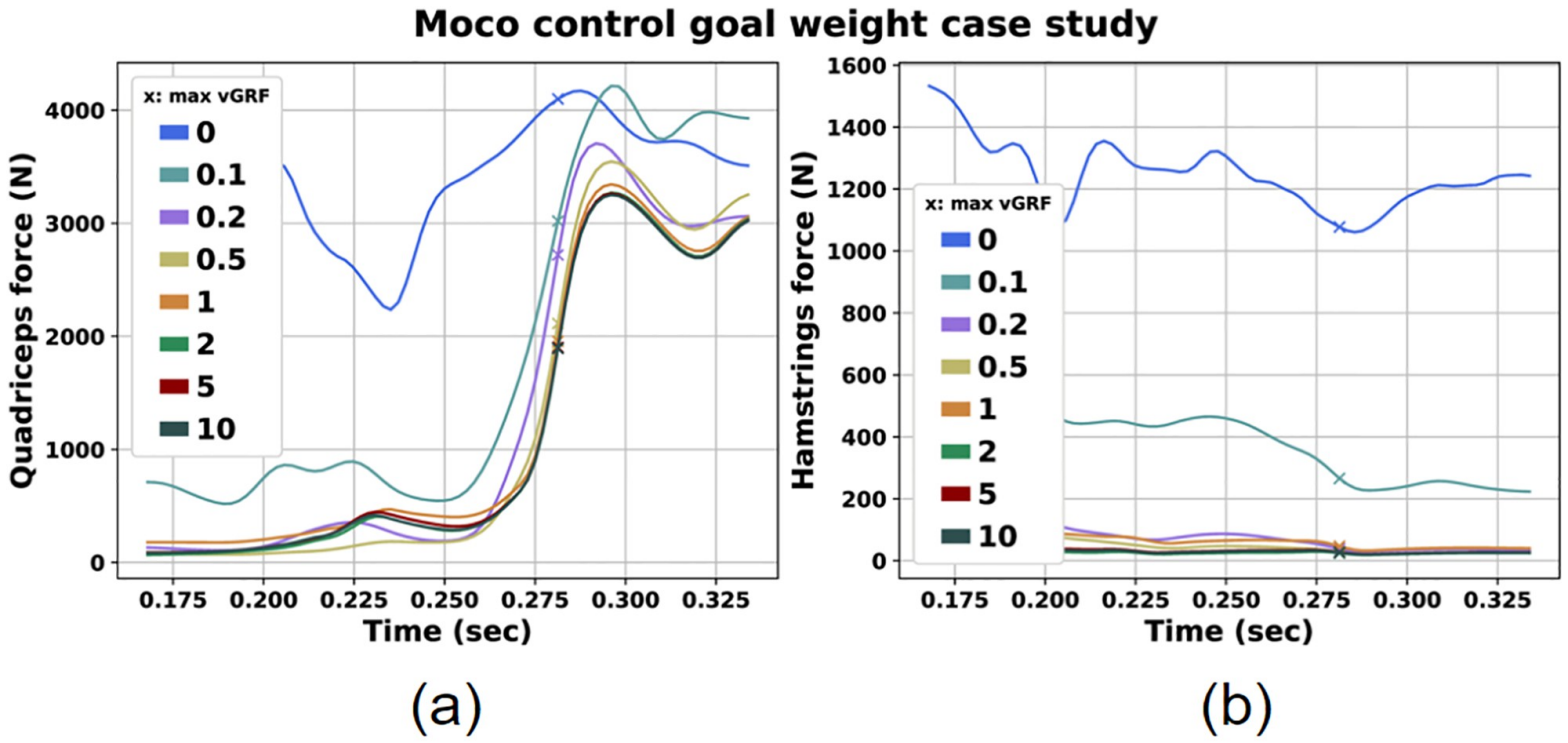
Quadriceps (a) and hamstrings (b) force for the Moco Control goal weight case study.

We observed that as we increased the weight of the effort goal, peak vGRF was also increased reaching a maximum value of 11.479 times BW for weight equal to 1. In general, for the upper limit weight values we noticed increased vGRF peaks. The same pattern was also spotted for the Q/H force ratio. In contrast, we observed that as the weight increased, AF decreased, although the differences between the peak values above the 0.1 weight are not very large. Regarding muscle forces, as it was expected for greater weights of the goal lower muscle forces were detected (Fig 10) since the objective function introduced to the problem aims to minimize the squared sum of the absolute value of the controls. Again, no significant differences were observed between the cases with weight greater than 0.1.

## Discussion

In this study, we developed an *in silico* simulation approach for predicting multiple single-leg landing motions and identifying ACL injury risk factors. Our aim was to develop a computational predictive framework that can be easily reproduced overcoming the inherent barriers of *in vivo* studies. To enhance the credibility of our adopted methodology we compare the ACL injury risk factors estimated by our predictive simulations with similar literature studies. The comparison is mainly qualitative focusing on variations of risk factor values for each case study. Nonetheless, we also quantitatively discuss our findings by comparing them to experimental or simulated arithmetic values for kJRF, kJRM, and GRF. As a general remark, we notice that our results are comparable with relative studies [26, 27, 40]. Additionally, when possible, we also highlight risk factor values that can potentially lead to an ACL injury, based on experimental studies. Although, we should mention that *in-vivo* knee joint load measurement during ACL injury would require sensor knee implants [58]. Thus, the reference studies that focus on ACL failure are conducted either *in-silico* or are video-based studies [59–61]. The reported values for AF and vGRF are in general similar to or lower than ours.

In this section, we discuss each case separately to identify common conclusions and arisen divergences concerning the different injury risk factors. Finally, limitations and possible future directions are also discussed.

### Landing height case

Starting from the landing height case, we concluded that vGRF, AF, and AbdM were larger as the height increased at the peak vGRF time instance. On the other hand, the opposite holds true for Q/H force ratio. These findings are in agreement with similar studies [26]. Mokhtarzadeh et al. examined single-leg landings from 30 and 60 cm of height and observed greater GRF peaks for landings from 60 cm [26]. More specifically, peak vGRF was 2.94 times BW when landing from 30 cm height and 3.83 when landing from 60 cm height. Nevertheless, in our study we observed lower peak values of vGRF ranging from 1.43 to 1.79 times BW for all examined landing heights (Fig 5 (a), Table 2). These vGRF values are close to those observed by Verniba et al. when examining single-leg landings from 22 and 44 cm heights [24]. Our values are lower compared to a video-based study examining ACL injury during landing in sports activities [61]. In any case, the GRF should be evaluated taking into account the muscle forces, as there is a correlation between these two parameters. This aspect will be discussed in the respective section (Moco Control goal weight case). Also, discrepancies could be related to the modified ground-foot contact model adopted in our study. In general, insight into foot-ground contact during landing motion is limited and a more detailed model might have captured more accurately the contact geometries that affect the predicted motion. This holds true for all the simulation scenarios we evaluated.

In addition, concerning AF we noted values ranging from 6.30 to 6.69 times BW. These values are close to the range of AF values detected by Mokhtarzadeh et al. [26]. More specifically, the reference AF values are around 4.4 and 6 times BW for landings from 30 and 60 cm respectively. In general, these values are greater than those reported in the literature for ACL failure [59, 60]. Moreover, we estimated AbdM values in the range 0.02–0.03 times BW. These are similar to studies examining landing conditions [62, 63].

Regarding Q/H force ratio, we detected that it was decreased as the initial landing height was increased at max vGRF. In agreement with our results, Mokhtarzadeh et al. stated that Q/H force ratio was lower for the landing from 60 cm compared with a landing from 30 cm. It should be mentioned that in our study we observed much higher values of Q/H force ratio at time of max vGRF for all examined scenarios. Again, differences between the studies could be due to the selected weight of the Moco effort goal as mentioned above.

The main outcome of the presented work regarding the landing height case is that as the landing height is increased, the risk of ACL injury is also incremented due to increased vGRF, AF and AbdM values at the peak vGRF moment. On the other hand, landings from lower heights (30, 35 cm) can limit the possibility of such injury as we observed lower values of vGRF, AF and AbdM.

### Hip rotation case

Additionally, we examined the influence of hip rotation in parameters associated with ACL injuries. Regarding internal hip rotation, we observed that in general vGRF, AF, and AbdM increased with larger hip rotations (Fig 6, Table 3). On the contrary, a range of hip external rotation between 10°— 15 demonstrated lower values for vGRF (Fig 7, Table 4). The estimated vGRF values are between 2.2 and 4.6 times BW. Also, we noted AF values between 3 and 4 times BW. These are in agreement with other research studies examining single-leg or double-leg landing motions [27, 28, 40]. In addition, we noted AbdM values close to those reported in literature [63, 64].

These findings are in general agreement with the literature, where a toe-out posture is associated with lower ACL injury risk. More specifically, the hip rotational alignment has been associated with knee valgus posture and ACL injuries. Yasuda et al. and Koga et al. studied landings from handball players where ACL injuries took place and observed that in the majority of landings with injury, the hip was internally rotated [35, 36]. Peel et al. compared landings with toe-in, toe-out and neutral position of the lower limb and concluded that the toe-out position could protect from injury while the toe-in position is associated with increased risk [38]. Summarizing, we could claim that the internally rotated hip position could possibly be associated with ACL injuries due to increased values in risk factors. On the contrary, the externally rotated hip position may be considered safer due to lower values in risk factors. Landing with 10° or 15° externally rotated hip can possibly be safer due to lower GRF.

### Trunk orientation case

Moreover, multiple studies have examined the influence of trunk orientation in the observed kinematics, GRF and kJRF during drop-landings. A leaning forward trunk has been associated with more flexion of the hip and knee joints, lower values of vGRF and lower mean amplitude of quadriceps EMG compared with an upright position during drop landings [39, 40]. Also, the upright position during landings has been linked with greater peak of vGRF, lower gastrocnemius and quadriceps activation and greater knee extensor moments [41]. In our study, we demonstrated that for all examined cases, landings that favored a forward leaning position resulted in lower risk of ACL injury. Specifically, we observed that when the trunk was leaning forward with 25° the peak vGRF was lower (Table 5). However, the lowest vGRF value was observed for the case of no flexion. This can be due to the similarity of the resulted motion with the initial guess given to the study. Also, for the case of 25° of trunk flexion, AbdM and IRM were lower compared to other angles of trunk flexion. Regarding landings with trunk leaning backwards, as a general remark, we observed greater vGRF compared with the upright position as the lumbar extension angle was increased (Table 6). Additionally, Q/H force ratio, CF, AbdM and IRM were greater at time of maximum vGRF for the cases of landings with trunk in backwards leaning. Also, Saito et al. observed greater knee valgus angles when landing in a trunk extension position compared with landings with trunk neutral position [42], which is in agreement with the AbdM values detected in our results. Concerning the trunk bending, Saito et al. observed that landings with the trunk leaning towards the opposite side of the landing leg or toward to the landing side led to greater values of knee valgus angles [42]. In our case, we observed that greater AbdM values occurred as the right or left bending angle was increased compared with the neutral position. This could lead to an increased knee valgus angle (Tables 7 and 8). Also, Jones et al. noticed greater GRF and knee joint loads when leaning towards the opposite direction of landing [65]. Similarly, in our study, we observed that the largest peak vGRF occurred when bending towards the right direction that is opposite to the landing leg with a value of 8.632 times BW.

Also we arithmetically compared our results to similar studies. We observed vGRF values from 1.3 to 8.6 times BW. On a similar note, Verniba et al. observed values from 1.2 to 2.7, while Niu et al. noted values in the range of 3–8 times BW [24, 27]. Also, in our case, the AF obtained values between 3.2 and 4.9, while Niu et al. observed values in the range of 2.9–5.2 [27]. All these values for AF are greater than those reported in cadaver studies simulating ACL failure during ski landing [59, 60]. Furthermore, the AbdM values were in the same range as those observed by Chappell et al. [64] with few exceptions. In more detail, we observed greater values than those reported for the following scenarios: 20° lumbar flexion, 10° lumbar extension, and 15°, 20°, 25° and 30° lumbar right bending.

### Permutations of knee joint agonists and antagonists muscles case

Furthermore, we investigated how modifying muscle forces for quadriceps and hamstrings affected the injury risk factors. An interesting observation was that the cases with strong quadriceps presented similar vGRF and AF values at peak vGRF time instance regardless of hamstrings strengthening or weakening as demonstrated in Table 10. Furthermore, we detected that the pairs with strong quadriceps exhibited the lowest vGRF values, while the AF values were greater compared to the other combinations (Fig 9, Table 10). Although both vGRF and AF are investigated as risk factors, we should emphasize that the AF is inherently and directly related to induced ACL loads [43]. On the other hand, vGRF acts in an indirect manner. As the muscles generate movement by producing forces, the foot applies a force to the ground and vice versa. The applied to the foot GRF, produces a joint reaction load to the ankle joint, that is further transmitted to the knee. This load contributes to the total knee joint reaction force in conjunction with other present loads produced by surrounding muscles and ligaments [44]. These forces further depend on kinematic conditions, such as the knee flexion angle [66]. Therefore, even if the vGRF seems lower for stronger quadriceps cases, which is also observed in similar studies [67], knee joint forces should also be considered, as they additionally depend on other present factors. In our case, larger quadriceps forces at peak vGRF time instance appear to contribute to the increase of the estimated AF, thus leading to increased ACL injury risk. This is in agreement with other published studies, particularly for knee flexion angles spanning from 0° (full extension) up to 60° [43, 68]. In our simulation studies, knee flexion angle at peak vGRF time instance is about 30° which is inside the aforementioned knee flexion angle range. Therefore, the increased quadriceps force can also be considered as an additional ACL injury risk factor [68, 69]. The greatest AF can be noticed for the case with both strong hamstrings and quadriceps (Table 10). This is in agreement with the study conducted by Morgan et al., where they observed greater quadriceps and hamstrings forces when the ACL was at higher injury risk [45].

In general, we estimated vGRF values between 0.6 and 1.4 times BW. These are lower than those observed by other studies [64]. Regarding AF we observed values similar to other studies examining single-leg landing motions [27, 28].

These observations clearly showcase the inherent complexity of the ACL injury mechanism and indicates how the muscles work in conjunction and the significance of the muscle redundancy issue. Nonetheless, our results highlight the impact of quadriceps force production on the vGRF and AF values, which are related to ACL injuries in single-leg landings.

### Moco control goal weight case

Finally, we investigated how the weight of the Moco effort goal (Eq 5) affects muscle activation and generated forces, and subsequently all landing and knee joint forces and moments. The objective of this study was to identify a weight factor that could lead to comparable GRF results with published studies. We should assert that this scenario was not conducted to investigate the impact of estimated risk factor values. Our intention was to acquire a baseline weight value that serves as an initial guess for subsequent predictions and lead to results that are comparable with similar experimental findings. Therefore, we compared the estimated vGRF for each weight value with GRF data presented in other studies assessing single-leg landings. Peak vGRF values between 2 and 8 times BW or greater can be found in literature [26, 28, 40]. In our study, we observed similar peak vGRF for goal weight ranging from 0 to 0.2.

### Limitations-future work

Summarizing, the fundamental objective of this work was to lay the background for a technical framework that can allow convenient setup of multiple case studies for evaluating single-leg landings conditions and their impact on certain risk factors of ACL injury. We applied the framework to answer several what-if scenarios of single-leg landing where the body posture, muscle forces and simulation settings were varied and the risk factors were evaluated. The results of this study evidently indicated the complexity of the ACL injury mechanism and its correlation with many risk factors. Thus, the phenomenon should be studied considering the association between these inherently different variables. The framework, as well as the simulation settings and results are made publicly available allowing for further modifications and meta-studies.

Of course, our work does not come without any limitations, especially considering the adopted modeling assumptions. First, we deployed musculoskeletal models that feature single DoF knee joints without modeling the contribution of other structures, such as cartilages and ligaments. Especially, we think that the presence of a suitable contact model between the femoral and tibial knee compartments would greatly improve detail and realism. A more detailed model might capture more accurately the contact geometries that affect the predicted motion. The same observation can be made for the ground-foot contact model and the adopted parameter values. Moreover, different population classes that feature varying anthropometric data could have been assessed.

All these limitations can serve as the foundation for future work to improve our current pipeline. Nonetheless, the presented work clearly showcased the promising potential of predictive simulations to evaluate multiple aspects of complicated phenomena, such as the ACL injury. This is supported by the findings of our study that are in general agreement with similar research works. Finally, we envision that further improvements can lead to a tool that can be used by physiotherapists and clinicians on adjusting rehabilitation and training plans based on subject-specific characteristics.

## Supporting information

S1 File Related supporting information. Simulation settings and implementation details.
(PDF)
