# Supplementary material for: Predictive simulation of single-leg landing scenarios for ACL injury risk factors evaluation
Source: PLoS One. 2023 Mar 9;18(3):e0282186. doi: 10.1371/journal.pone.0282186 (PMC9997920; doi:10.1371/journal.pone.0282186)
Supplement: S1 File — Simulation settings and implementation details. (PDF) [file pone.0282186.s001.pdf]

Predictive simulation of single-leg landing scenarios for ACL injury risk factors evaluation

Evgenia Moustridi[1*], Konstantinos Risvas[1], Konstantinos Moustakas[1]

1 Department of Electrical and Computer Engineering, University of Patras, Patras, Achaia, Greece

* evgmoustridi@ece.upatras.gr (EM)

Supplementary material

1. FOOT-GROUND CONTACT

In this section we present selected parameters of the foot-ground contact model used for the simulations. The values were assigned based on other research studies [1] and are presented in S1 Table.

Parameter	Value
Stiffness	10^7 (N/m^2)
Dissipation	0.50 (s/m)
Static Friction	0.90
Dynamic Friction	0.90
Viscous Friction	0.59
Transition Velocity	0.20 (m/s)

S1 Table. Parameters for the contact model.
Table notes the parameters of the contact model used for the simulation.

2. IMPLEMENTATION

The source code along with any related material for this publication are publicly available, providing scripts so that the readers can comprehend, reuse and reproduce the results of all the examined cases. Also, the resulted motion files and exported plots are all included. The following open source frameworks were used for the simulations: OpenSim, SCONE and Moco tool. All scripts are implemented using python.

The experiments were conducted using a computer machine with an i7-9700 Intel(R) Core(TM) processor @3.00 GHz, a memory of 16GB, and a Windows 10 64bit operating system. Every simulation scenario required about 45 to 60 minutes to complete regarding the above specifications (except for the muscle force case study which required about 60 to 120 minutes per study).

A. Predict motion with SCONE

In this subsection, we present a general overview of the simulation setup and its basic components for predicting a single-leg landing motion in SCONE (in lua script).

```
Optimizer {
  # definition of model used in simulation
  OpenSimModel { }
  Controller {
    type = ReflexController
    # Muscle length reflexes
    MuscleReflex {}
    # Vestibular reflexes
    BodyPointReflex {}
  CompositeMeasure {
```

```
      # measure to minimise GRF
      ReactionForceMeasure {}
      # measure for not falling
      BalanceMeasure {}
      # measute for desired joint ranges
      DofMeasure {}
   }
}
```

As it can be noted, the Optimizer contains the musculoskeletal model at its initial state, a reflex controller and a composite measure. The reflex controller includes "MuscleReflex" entries that simulate proprioceptic reflexes and a "BodyPointReflex" that simulate the vestibular reflexes.

The composite measure contains the following measures:

- **A parameter that checks if the model falls below a specified value**
- **A penalty that minimizes the Ground Reaction Forces (GRF) (ReactionForceMeasure)**
- **Penalties for exceeding desired joint ranges (DofMeasure)**. The desired joint range for the ankle was: [25°, 35°] (positive dorsiflexion). For the knee was: [$-60°$, $-30°$] (positive flexion) and for the pelvis tilt was: [$-50°$, 0°].

Penalties are applied when these measures are violated.

B. Simulations with Moco

Operations on the selected musculoskeletal models are valid for all the simulations in Moco (tracking and predictive) and are presented along with simulation setup steps in Moco.

B.1. Model operations

For all the simulations conducted in Moco (tracking and predictive), certain modifications were applied to the musculoskeletal model. The muscle model was selected as the "DeGrooteFregly" muscle type, since this model is compliant with Moco. The passive fiber forces were ignored and the active fiber force width for all muscles was scaled with a factor of 1.5. Ideal torque actuators were appended to the Degrees of Freedom (DoFs) that were not actuated by muscles. Also, reserve actuators were added to all DoFs to act supplementary to the already present muscles. The maximum torque was set to 100 Nm. All these operations are demonstrated next:

```
    model = osim.ModelProcessor("Gait2392.osim")
    # turn off tendon compliance
    model.append(osim.ModOpIgnoreTendonCompliance())
    # replace with "DeGrooteFregly" muscle type
    model.append(osim.ModOpReplaceMusclesWithDeGrooteFregly2016())
    # turn off passive fiber forces
    model.append(osim.ModOpIgnorePassiveFiberForcesDGF())
    # Scale the active fiber force curve width
    model.append(osim.ModOpScaleActiveFiberForceCurveWidthDGF(1.5))
    # add reserve actuators to the model
    model.append(osim.ModOpAddReserves(100))
```

C. Track with Moco

Regarding the two tracking studies in Moco, the pipeline was similar, except from the musculoskeletal models. The simulation initial and final time were identical to these of the SCONE trajectory. The track tool instance was connected to the problem. Instantly the "MocoStateTrackingGoal" was added to the tracking study. Some states of the model were edited to further assist the trajectory solution. The bounds for these DoFs were set based on the initial and final states of the predicted motion from SCONE simulation. A detailed overview of these bounds is presented in S2 Table. For the DoFs that are not included in this table no bounds were set. Moreover, we applied bounds for the initial state and the entire motion for the activation of all muscles. At the first time instant the activation of all muscles was set to zero. Furthermore, both models consists of identical DoFs and the following description concerns both of them. An overview of the commands that were used to setup the analysis is presented next:

```
    # define musculoskeletal model
    model = osim.ModelProcessor()
    # set MocoTrack tool
    track = osim.MocoTrack()
    # define model
    track.setModel()
```

```
    # set table containing values of model state variables
    track.setStatesReference()
    # set initial time
    track.set_initial_time()
    # set final time
    track.set_final_time()
    # initialize MocoTrack in the study
    study = track.initialize()
    # access  MocoProblem within the study.
    problem = study.updProblem()
    problem.setStateInfo()
    # initialize the solver
    solver = study.initCasADiSolver()
    # set the initial guess motion file
    solver.setGuess('bounds')
    solution = study.solve()
```

DoFs	Bounds	Initial Bounds	Final Bounds
pelvis_ty	(0.7, 1.25)	1.25	(0.75, 0.85)
pelvis_†	(-0.01, 0.01)	0	-
lumbar_†	(-0.01, 0.01)	0	-
hip_flexion_l	(0.08, 0.5)	0.087	-
hip_†	(-0.01, 0.01)	0	-
subtalar_angle‡	(-0.01, 0.01)	0	-
mtp_angle‡	(-0.01, 0.01)	0	-

† This stands for all DoFs of the joint if they are not explicitly defined.
‡ This stands for both right and left joints.

S2 Table. Bounds for the DoFs of Gait2354 and Gait2392 OpenSim models for MOCO Track tool.

D. Predict motion with Moco

Regarding prediction in Moco, we created a new study for each investigated scenario. The main simulation setup which is valid for all the studies is presented in this subsection. Specific settings for each case study will be presented in the following sections. The common setting for all cases was the initial guess which was set as the MocoTrack output.

```
    # define musculoskeletal model
    model = osim.ModelProcessor()
    # set MocoStudy
    study = osim.MocoStudy()
    # access  MocoProblem within the study.
    problem = study.updProblem()
    #Set the model
    problem.setModel()
    #Set time bounds
    problem.setTimeBounds()
    # set bounds for state variables
    problem.setStateInfo()
    # add goals of interest
    problem.addGoal()
    # initialize the solver
    solver = study.initCasADiSolver()
    # set the initial guess motion file
    solver.setGuessFile()
    solution = study.solve()
```

D.1. Initial height case study

In this section, we describe the simulation setup of drop-landing from different initial heights. The model used was "Gait2392". The pelvis joint vertical position value was modified in order to achieve landings from 30, 35, 40, 45, 50 and 55 cm of height. For every height value a new study

was created with the parameters described previously. The solution acquired with the track tool was used as an initial guess for the solver. Also, the "MocoControlGoal" or effort goal was added to the problem with a weight of 0.001.

Apart from the pelvis joint vertical position value which was adjusted in order to achieve multiple initial landing heights, all the other DoFs remained identical for the initial state in all scenarios. It should be mentioned that a deviation of 0.01 cm of the selected landing height was allowed in all cases, as with all DoFs of the model.

D.2. Hip rotation case study

Again, we used the "Gait2392" OpenSim model, and the previously tracked solution was used as an initial guess for the solver. Also, the "MocoControlGoal" was added to the problem with a weight of 0.001. In S3 Table we display the bounds assigned to all DoFs in the Moco studies. For *hip_adduction* of the left lower limb we did not assign bounds because it is highly related to *hip_rotation* and we wanted to examine how it will respond to different conditions of hip rotation.

DoFs	Initial bounds	Final bounds	Bounds
pelvis_ty	1.25	(0.75, 0.85)	(0.7, 1.25)
pelvis_[†]	0	-	(-0.57, 0.57)
hip_flexion_r	30°	-	(29°,30°)
hip_rotation_r	0°	-	(−0.01°,0.01°)
hip_adduction_r	0°	-	(−0.01°,0.01°)
knee_angle_r	−120°	-	(−121°,−119°)
ankle_angle_r	0°	-	(−0.57°, 0.57°)
subtalar_angle_r	0°	-	(−0.57°, 0.57°)
mtp_angle_r	0°	-	(−0.57°,0.57°)
hip_flexion_l	5°	-	(4.5°,28.5°)
hip_rotation_l	-	-	(v-0.57°,v+0.57°)
hip_adduction_l	-	-	-
knee_angle_l	−11.5°	-	(−57°,0°)
ankle_angle_l	−34°	-	(−34°,45°)
subtalar_angle_l	-	-	(−2.85°,2.85°)
mtp_angle_l	0°	-	(−5.73°, 5.73°)

[†] This stands for all DoFs of the joint if they are not explicitly defined.
[‡] v is the value of the generalized coordinate under consideration for each scenario.

S3 Table. Values for DoFs of the model for different values of left hip rotation scenarios

D.3. Trunk orientation case study

Regarding the trunk orientation case study, different studies were produced using the initial guess described previously and certain bounds for the initial and final states that are presented in S5 Table, S4 Table. The musculoskeletal model was "Gait2392" and the "MocoControlGoal" or effort goal was added to the problem with a weight of 0.002.

DoFs	Angle (degrees)							Bounds
lumbar_flexion	0	5	10	15	20	25	30	(v-0.57,v+0.57)†
lumbar_extension	0	5	10	15	20	-	-	"
lumbar_bending	0	5	10	15	20	25	30	"

† v is the value of the generalized coordinate under consideration for each scenario.

S4 Table. Overview of investigated values for lumbar joint DoFs. We also describe the bounds for each value.

DoFs	Initial bounds	Final bounds	Bounds
pelvis_ty	1.25	(0.75, 0.85)	(0.7, 1.25)
pelvis†	0°	-	(−0.57°, 0.57°)
hip_flexion_r	30°	-	(29°,30°)
hip_rotation_r	0°	-	(−0.01°,0.01°)
hip_adduction_r	0°	-	(0.01°,0.01°)
knee_angle_r	−120°	-	(−121°,−119°)
ankle_angle_r	0°	-	(−0.57°, 0.57°)
subtalar_angle_r	0°	-	(−0.57°, 0.57°)
mtp_angle_r	0°	-	(−0.57°, 0.57°)
hip_flexion_l	5°	-	(4.5°,28.5°)
hip_rotation_l	0°	-	(−0.01°, 0.01°)
hip_adduction_l	0°	-	(−0.01°, 0.01°)
knee_angle_l	−11.5°	-	(−57°,0°)
ankle_angle_l	−34°	-	(−34°,45°)
subtalar_angle_l	-	-	(−2.86°,2.86°)
mtp_angle_l	0°	-	(−0.57°, 0.57°)

† This stands for all DoFs of the joint if they are not explicitly defined.

S5 Table. Bounds for the DoFs of the model for the trunk orientation case study.

D.4. Muscle force of knee joint agonists and antagonists case study

For the muscle forces case study, the model used was "Gait2392" and the effort goal was added to the problem with a weight of 0.

The quadriceps muscles include rectus femoris, vastus medialis, vastus lateralis and vastus intermedialis. Hamstrings muscle group combines the semimembranosus, semitendinosous, biceps femoris long head and biceps femoris short head. In S6 Table we display the value of the standard max isometric force fore each muscle, along with the value when it is weakened or strengthened. Based on S6 Table, nine cases were simulated with different combinations of normal, weak and strong muscles. These cases are demonstrated in S7 Table.

Muscle	max Isometric force	Weak	Strong
Rectus femoris	1169	760	1578
Vastus intermedialis	1365	887	1842
Vastus lateralis	1871	1216	2526
Vastus medialis	1294	841	1746
Semimembranosus	1288	837	1739
Semitendinosus	410	267	554
Bicep femoris long head	896	582	1210
Bicep femoris short head	804	523	1085

S6 Table. Values for max isometric force for all muscles in hamstrings ans gastrocnemius muscle groups. Also, the modified max isometric forces for these muscles for their strengthening or weakening are included.

The bounds used for the initial and final states were identical to those used in previous cases for the right lower limb. The trunk and the left lower limb DoFs were restricted based on the initial guess and small deviations were allowed. A "MocoControlGoal" was used with a weight of 0.

case	Quadriceps	Hamstrings
1	strong	strong
2	strong	normal
3	strong	weak
4	normal	strong
5	normal	normal
6	normal	weak
7	weak	strong
8	weak	normal
9	weak	weak

S7 Table. Demonstration of the nine cases with different combinations of normal, weak and strong muscles.

D.5. Moco Control goal weight case study

For the Moco Control goal weight case study, we examined the following effort goal weight values: 0, 0.1, 0.2, 0.5, 1, 2, 5 and 10. Again, the selected model was the "Gait2392", and the tracked solution was the initial guess. Also, the DoFs were bounded as described in previous sections. All parameters were identical for all cases except for the goal weight.

3. GRID CONVERGENCE STUDY

A Grid convergence study was conducted. We run the simulation several times with different grid densities. The grid of "mesh" in Moco stands for the discritization of the movement in time. This, defines the timestep that separates the nodes at which the problem is evaluated. We conducted the same simulation for the following numbers of nodes: 11, 21, 51, 101, 201, 301, 401, 501, 601, 701 and 801. The resulted values for the number of iterations, simulation time and cost function are demonstrated in S8 Table.

Intervals	Nodes	Iterations	Simulation Time(h)	Cost function(xe4)
5	11	134	0.06	4.868
10	21	67	0.096	5.354
25	51	62	0.218	14.882
50	101	45	0.258	23.168
100	201	43	0.375	30.959
150	301	50	0.627	30.973
200	401	52	0.897	30.967
250	501	52	1.097	30.983
300	601	57	1.481	30.989
350	701	56	1.652	30.985
400	801	57	1.935	30.983

S8 Table. The number of iterations, the simulation time and the value of the cost function for different number of intervals.

As it can be observed from S8 Table, increasing the number of intervals results in greater simulation time and greater values for the cost function. Though, we can note that for number of intervals greater than 100 the increasement in the cost function is very small. For that reason, in all predictive simulations in Moco we used 100 intervals.

REFERENCES

1. Serrancoli G, Falisse A, Dembia C, Vantilt J, Tanghe K, Lefeber D, et al. Subject-Exoskeleton Contact Model Calibration Leads to Accurate Interaction Force Predictions. IEEE Trans Neural Syst Rehabil Eng. 2019;27: 1597–1605. doi:10.1109/TNSRE.2019.2924536
